# Induction of G1 and G2/M cell cycle arrests by the dietary compound 3,3'-diindolylmethane in HT-29 human colon cancer cells

**DOI:** 10.1186/1471-230X-9-39

**Published:** 2009-05-29

**Authors:** Hyun Ju Choi, Do Young Lim, Jung Han Yoon Park

**Affiliations:** 1 Department of Food Science and Nutrition, Hallym University, Chuncheon, 200-702, Republic of Korea

## Abstract

**Background:**

3,3'-Diindolylmethane (DIM), an indole derivative produced in the stomach after the consumption of broccoli and other cruciferous vegetables, has been demonstrated to exert anti-cancer effects in both *in vivo *and *in vitro *models. We have previously determined that DIM (0 – 30 μmol/L) inhibited the growth of HT-29 human colon cancer cells in a concentration-dependent fashion. In this study, we evaluated the effects of DIM on cell cycle progression in HT-29 cells.

**Methods:**

HT-29 cells were cultured with various concentrations of DIM (0 – 30 μmol/L) and the DNA was stained with propidium iodide, followed by flow cytometric analysis. [^3^H]Thymidine incorporation assays, Western blot analyses, immunoprecipitation and *in vitro *kinase assays for cyclin-dependent kinase (CDK) and cell division cycle (CDC)2 were conducted.

**Results:**

The percentages of cells in the G1 and G2/M phases were dose-dependently increased and the percentages of cells in S phase were reduced within 12 h in DIM-treated cells. DIM also reduced DNA synthesis in a dose-dependent fashion. DIM markedly reduced CDK2 activity and the levels of phosphorylated retinoblastoma proteins (Rb) and E2F-1, and also increased the levels of hypophosphorylated Rb. DIM reduced the protein levels of cyclin A, D1, and CDK4. DIM also increased the protein levels of CDK inhibitors, p21^CIP1/WAF1 ^and p27^KIPI^. In addition, DIM reduced the activity of CDC2 and the levels of CDC25C phosphatase and cyclin B1.

**Conclusion:**

Here, we have demonstrated that DIM induces G1 and G2/M phase cell cycle arrest in HT-29 cells, and this effect may be mediated by reduced CDK activity.

## Background

Epidemiologic data continue to support the hypothesis that the intake of *Brassica *plants, including turnips, kale, broccoli, cabbage, Brussels sprouts, and cauliflower, may exert protective effects against various types of cancers [1-4]. Dietary glucosinolates present in *Brassica *species have been previously shown to protect against several types of cancer [5,6]. Indole-3-carbinol (I3C) is the principal hydrolysis product of the glucosinolate glucobrassicin [7], and has been shown to offer significant protection against cancer in animal models induced by a variety of chemical carcinogens [8-10], as well as in cultured human cancer cells [11-13]. Initial clinical trials in women have shown that I3C may prove to be a promising agent against cervical and breast cancers (reviewed in [14]).

I3C is chemically unstable in the low pH environment of the stomach, and has been demonstrated to undergo self-condensation reactions, resulting in the production of a variety of polymeric products. Among them, 3,3'-diindolylmethane (DIM) is a principal product, which is acid-stable [15,16] and is measurable in both human urine samples [17] and animal tissues [18]. It has been shown that DIM reduces carcinogenesis in a variety of animal cancer models, including the tumor growth of injected TRAMP-C2, a mouse prostate cell line, in C57BL/6 mice [19], 7,12-dimethylbenz [a]anthracene (DMBA)-induced mammary tumors in rats [20], benzo [a]pyrene-induced stomach cancers in mice, and the growth of transplantable human breast carcinoma and angiogenesis in mice [21]. In addition, results generated with *in vitro *cell culture studies have shown that DIM inhibits the proliferation of a variety of cancer cell types, including prostate (Reviewed in [22]) and breast [23,24] cancer cells, via the induction of cell cycle arrest and apoptosis.

Carcinogenesis is a multistep process, and there are several opportunities for intervention to halt, regress, or delay the carcinogenic process. One of these anti-carcinogenic actions involves the inhibition of cell cycle progression, because insensitivity to growth-inhibitory (antigrowth) signals is one of the hallmarks of cancer [25]. Therefore, searching for bioactive food components with the ability to inhibit cancer cell cycle progression should facilitate the identification of new compounds that can potentially inhibit cancer progression.

Cell cycle progression depends on the activation of cyclin-dependent kinases (CDK), which act consecutively in G1 to initiate S phase and in G2 to initiate mitosis. Upon mitogenic stimuli, D-type cyclins are induced and bind to and activate CDK4 and CDK6. The cyclin D-dependent kinases initiate the phosphorylation of retinoblastoma proteins (Rb), relieving E2F from negative restraints and allowing for the expression of certain E2F-target genes. Cyclin E-CDK2 completes Rb phosphorylation, further permitting the activation of E2F-responsive genes. CDK2 is also capable of binding to A-type cyclins during the S phase, whereas the control of G2 and M phases depends principally upon the cyclin A-cell division cycle (CDC)2 and cyclin B-CDC2 (reviewed in [26,27]). Several mammalian CDK inhibitors (CDKIs) have been previously identified. One group is the INK4 (inhibitors of CDK4) family, which has four members–p16^INK4a^, p15^INK4b^, p18^INK4c ^and p19^INK4d^–all of which share the ability to control the G1/S transition [28,29]. The second group of CDKIs includes p21^CIP1/WAF1 ^and p27^KIPI^. These proteins conduct pivotal functions in cell cycle regulation via the coordination of internal and external signals that inhibit cell cycle progression at critical checkpoints [29].

There have been only a few studies conducted thus far that have assessed the effects of DIM on the prevention of colon cancer. DIM has been shown to effect a dose-dependent cytotoxicity in HT-29 human colon adenocarcinoma cells [30]. We have previously demonstrated that DIM inhibits the growth of HT-29 and HCT-116 human colon cancer cells and induced apoptosis in these cells [31]. To the best of our knowledge, the molecular mechanisms by which DIM inhibits the cell cycle progression of colon cancer cells have yet to be clearly elucidated. The results of this study show that DIM delays cell cycle progression at the G1 and G2/M phases and inhibits the activity of CDK2 and CDC2 in HT-29 cells.

## Methods

### Materials

The following reagents and chemicals were obtained from the indicated suppliers: DIM (LKT Laboratories, St. Paul, MN); anti-β-actin and p16^INK/CDKN2 ^(Sigma, St. Louis, MO); antibodies against CDC25C, cyclin B1, and phospho-Rb (Cell Signaling, Danvers, MA); horseradish peroxidase (HRP)-conjugated anti-rabbit and anti-mouse IgG and protein A-Sepharose (GE Healthcare Biosciences, Piscataway, NJ); [γ-^32^P]ATP (Perkin Elmer Life Science, Waltham, MA); anti-cyclin D1 antibody (Neomarkers, Fremont, CA); and antibodies against p21^CIP1/WAF1^, p27^KIPI^, cyclin A, cyclin E, CDK2, CDK4, CDC2, E2F-1, and Rb (Santa Cruz Biotechnology, Santa Cruz, CA).

### Cell culture

The HT-29 cell line was obtained from the American Type Culture Collection (ATCC, Manassas, VA) and maintained in DMEM/F-12 supplemented with 10% FBS, 100,000 U/L of penicillin, and 100 mg/L of streptomycin. In order to determine the effects of DIM, we plated the cells with DMEM/F12 containing 10% FBS. Prior to DIM treatment, the cell monolayers were rinsed and serum-deprived for 24 h with DMEM/F-12 containing 1% FBS. After serum deprivation, we replaced the medium with fresh serum deprivation medium with or without 30 μmol/L of DIM.

To evaluate [^3^H]thymidine incorporation, HT-29 cells were plated in 96-well plates at a density of 6,000 cells/well, serum-deprived, and treated with various concentrations of DIM for 9 h, as described above. 0.5 μCi [^3^H]thymidine was then added to each well, and the incubation continued for an additional 3 h at 37°C. The incorporation of [^3^H]thymidine into the DNA of HT-29 cells was determined as previously described [32].

### Cell cycle analysis by flow cytometry

Cells were plated in 100 mm dishes at 2,000,000 cells/dish in DMEM/F-12 containing 10% FBS. The cells were serum-deprived and treated for 12 h with various concentrations of DIM, then trypsinized. The nuclei were stained with propidium iodide as described previously [33] and subjected to fluorescence-activated cell sorting analysis utilizing the FACScan system (Becton Dickinson, Franklin Lakes, NJ). The data were analyzed using ModFit V.1.2. software.

### Immunoprecipitation and immunoblot analyses

The cells were lysed as previously described [34] and the protein concentrations of lysates were determined using a bicinchoninic acid protein assay kit (Thermo Fisher Scientific, Rockford, IL). For immunoprecipitation, cell lysates (750 μg protein) were precleared with 1.5 μg of normal rabbit IgG and 100 μL of protein A-Sepharose bead slurry on a rotating platform for 1 h, then centrifuged for 10 min at 12,000 rpm at 4°C. The supernatants were incubated for 1 h with 1 μg of anti-CDK2 or anti-CDC2 antibody at 4°C. The protein A-Sepharose was added and incubated for an additional 1 h at 4°. The beads were then washed four times in lysis buffer via 5 min of centrifugation at 2,500 rpm at 4°C. Total cell lysates (50 μg protein) or immunoprecipitated proteins were resolved on SDS-PAGE (4 – 20% or 10 – 20%) and transferred onto a polyvinylidene fluoride membrane (Millipore, Billerica, MA). The blots were incubated for 1 h with anti-CDK2 (1:1000), anti-CDK4 (1:1000), anti-cyclin A (1:1000), anti-cyclin D1 (1:200), anti-cyclin E (1:1000), anti-p21^CIP1/WAF1 ^(1:1000), anti-p27^KIP1^(1:1000), anti-phospho-Rb (1:1000), anti-Rb (1:1000), anti-E2F-1 (1:1000), or anti-β-actin (1:2000) antibody. The membranes were then incubated with anti-mouse or anti-rabbit HRP-conjugated antibody. The signal was detected with a chemiluminescence detection system (Millipore). Densitometric analysis was conducted using the Bio-profile Bio-ID application (Vilber-Lourmat, Marne-la-Vallée, France). Expression levels were normalized to β-actin and the control (0 μmol/L DIM at 2 h) levels were set at 100%.

### Reverse transcription polymerase chain reaction (RT-PCR)

Total RNA was isolated using Tri reagent (Sigma), and cDNA was synthesized using 2 μg of total RNA with SuperScript™ II reverse transcriptase (Invitrogen, Carlsbad, CA), as described previously [35]. For cDNA amplification, primers for human p21^CIP1/WAF1 ^(upstream primer; 5'-AAR AAG GAA GCG ACC TGC AA-3'; downstream primer, 5'-CCA ACG CTT TTA GAG GCA GA-3', annealing at 55°C for 1 min with 38 cycles), p27^KIP1 ^(upstream primer; 5'-AAT AAG GAA GCG ACC TGC AA-3'; downstream primer, 5'-CAA ACG CTT TTA GAG GCA GA-3', annealing at 60°C for 1 min with 27 cycles), and p16^INK/CDKN2 ^(upstream primer; 5'-GCC CAA CGC ACC GAA TAG-3'; downstream primer, 5'-ACG GGT CGG GTG AGA GTG-3', annealing at 54°C for 1 min with 37 cycles) were used. The expressions of human β-actin transcripts were assessed as an internal control, as described previously [34]. For each combination of primers, the kinetics of the PCR amplification were studied, the number of cycles corresponding to the plateau was determined, and the PCR was conducted within the exponential range. The PCR products were then separated in 1 or 2% agarose gel and stained with ethidium bromide. Bands corresponding to each specific PCR product were quantified via the densitometric scanning of the exposed film using the Bio-profile Bio-ID application.

### *In vitro *kinase assay

Cell lysates (750 μg protein) were immunoprecipitated with polyclonal antibody against CDK2 or CDC2 as described above. After capture with protein A-Sepharose and subsequent washes, the beads were incubated for 30 min at 37°C in 15 μL of kinase buffer [36] containing 2 μg of histone H1 (Roche, Nutley, NJ) and 3 μCi of [γ-^32^P]ATP (CDK2 kinase assay) or in 30 μL of kinase buffer containing 2 μg of histone H1 and 5 μCi of [γ-^32^P]ATP (CDC2 kinase assay). The reaction was halted by boiling the samples for 5 min in SDS sample buffer. The ^32^P-labeled histone H1 was resolved on SDS-PAGE, and the gels were dried and subjected to autoradiography. Signals were quantified via densitometric scanning of the exposed film.

### Statistical analysis

The results were expressed as the means ± SEM for the indicated number of separate experiments. Statistical data analysis was conducted with ANOVA. Differences between the treatment groups were analyzed via Duncan's multiple range test or Student's t-test. Differences were considered significant at *P *< 0.05. Statistical analyses were conducted using the SAS system for Windows, version 8.12.

## Results

### DIM induces cell cycle arrest at the G1 and G2/M phases and inhibits DNA synthesis in HT-29 cells

**Figure 1:**
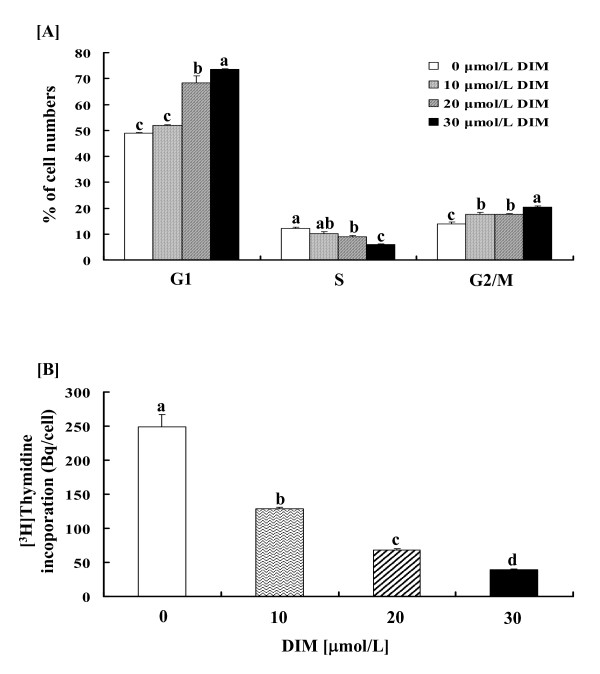
**DIM induces cell cycle arrest at G1 or G2/M phases and inhibits [^3^H]thymidine incorporation in HT-29 cells**. (A) Cells were plated in 6-well plates at 200,000 cells/well with DMEM/F12 supplemented with 10% FBS. 24 h after plating, the monolayers were serum-deprived for 24 h with DMEM/F-12 containing 1% FBS. After serum deprivation, cells were incubated for 12 h in serum deprivation medium containing 10, 20 or 30 μmol/L DIM. The nuclei were stained with propidium iodide and the cell cycle was analyzed via flow cytometry. (B) Cells were plated at a density of 6,000 cells/well in 96-well plates and serum-deprived, then incubated for 9 h in serum deprivation medium containing 0, 10, 20 or 30 μmol/L DIM. [^3^H]Thymidine was added, and the cells were incubated for an additional 3 h to measure the incorporation into DNA. Each bar represents the mean ± SEM (n = 6). Comparisons between groups that yielded significant differences (*P *< 0.05) are indicated by different letters above each bar.

We have previously shown that DIM reduces the numbers of viable HT-29 cells in a dose-dependent manner (10 – 30 μmol/L) [31]. We have also reported that these concentrations of DIM do not affect the viability of IEC-6 cells, which are normal small intestinal epithelial cells [31]. To determine whether DIM regulates cell cycle progression in HT-29 cells, the cells were treated for 12 h with 10 – 30 μmol/L of DIM, and the DNA was stained with propidium iodide, followed by FACS analysis. The treatment of cells with various concentrations of DIM for 12 h resulted in dose-dependent increases in the percentage of cells in G1 and G2/M phases with a concomitant reduction in cell numbers in the S phase (Fig. 1A). However, the differences between 0 and 10 μmol/L of DIM in G1 and S phase were not statistically significant. Consistent with the occurrence of cell cycle G1 arrest at 12 h, DIM reduced the [^3^H]thymidine incorporation into the DNA of HT-29 cells in a dose-dependent manner (Fig. 1B).

### DIM reduces the levels of CDK4, cyclin A, and cyclin D1 proteins

**Figure 2:**
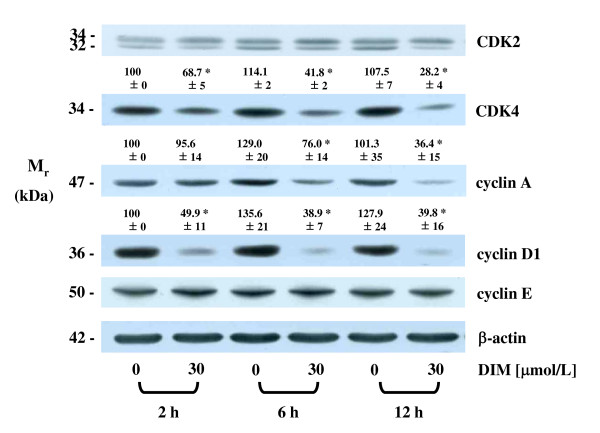
**DIM reduces the levels of CDK4 and of cyclin A and E in HT-29 cells**. Cells were treated with DIM as described in Figure 1A. Cell lysates were analyzed via Western blotting with the indicated antibodies. Photographs of the chemiluminescent detection of the blots, which were representative of three independent experiments, are shown. The relative abundance of each band to its own β-actin was quantified, and the control levels were set at 100%. The adjusted mean ± SEM (n = 3) of each band is shown above each blot. *Significantly different from 0 μmol/L DIM, *P <*0.05.

We first examined the effects of DIM on proteins controlling the G1/S phase transition. DIM did not affect the levels of the CDK2 protein, whereas the levels of CDK4 were reduced in HT-29 cells within 2 h after treatment with 30 μmol/L DIM. The levels of cyclin A and cyclin D1 were reduced within 6 h and 2 h, respectively, after treatment with 30 μmol/L of DIM, whereas the levels of cyclin E were not altered by DIM treatment (Fig. 2).

### DIM increases the levels of p21 and p27 proteins and mRNAs

**Figure 3:**
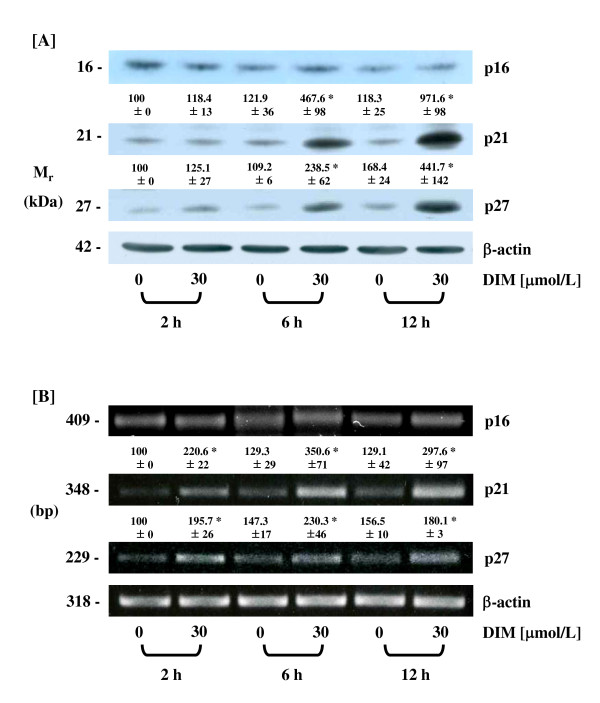
**DIM increases the levels of p21 and p27 proteins and mRNAs in HT-29 cells**. Cells were treated with different concentrations of DIM as described in Figure 1. (A) Cell lysates were analyzed by Western blotting with the indicated antibodies. Photographs of the chemiluminescent detection of the blots, which were representative of three independent experiments, are shown. (B) Total RNA was isolated and reverse-transcribed, and PCR was performed with the specific primers for p16, p21, p27, or β-actin. PCR products were separated in an agarose gel with TAE buffer and stained with ethidium bromide. Photographs of the ethidium bromide-stained gels, which are representative of three independent experiments, are shown. The relative abundance of each band to its own β-actin was quantified, and the control levels were set at 100%. The adjusted mean ± SEM (n = 3) of each band is shown above each blot. *Significantly different from 0 μmol/L DIM, *P *< 0.05.

Because the CDK inhibitors p21^CIP1/WAF1 ^and p27^KIP1 ^are known to inhibit CDK activity, we subsequently examined the effects of DIM on the levels of p21^CIP1/WAF1 ^and p27^KIP1 ^proteins. The levels of p21^CIP1/WAF1 ^and p27^KIP1 ^proteins were markedly increased in HT-29 cells at 6 h after treatment with 30 μmol/L of DIM (Fig. 3A). In order to determine whether DIM regulates the levels of p21^CIP1/WAF1^and p27^KIP1 ^at the RNA level, we conducted RT-PCR analyses. The mRNA levels of p21^CIP1/WAF1 ^and p27^KIP1 ^increased within 2 h after treatment with 30 μmol/L of DIM, which were consistently higher during the 12 h of DIM treatment (Fig. 3B). We also examined the effects of DIM on the levels of p16^INK/CDKN2 ^(also known as p16^INK4a^) protein and mRNA. The levels of p16^INK/CDKN2 ^protein and mRNA were not affected by DIM treatment (Fig. 3A and 3B).

### DIM inhibits the activity of CDK2 and the levels of phospho-Rb and increases those of hypophosphorylated Rb

**Figure 4:**
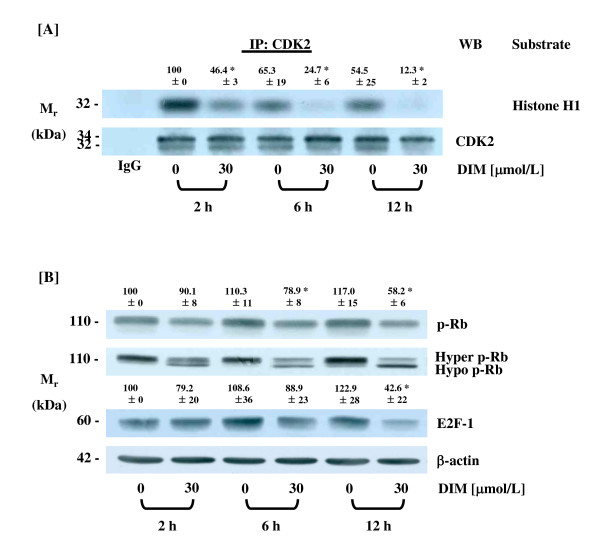
**DIM inhibits CDK2 activity and reduces the levels of phospho-Rb and E2F-1 in HT-29 cells**. Cells were treated with different concentrations of DIM as described in Figure 1. (A) CDK2 was immunoprecipitated and analyzed via an *in vitro *kinase assay using histone H1 as a substrate. An autoradiograph of the dried gel, which is representative of three independent experiments, is shown. (B) Cell lysates were analyzed via Western blotting. Photographs of the chemiluminescent detection of the blots, which were representative of three independent experiments, are shown. The relative abundance of each band to its own CDK2 (A) or β-actin (B) was quantified, and the control levels were set at 100%. The adjusted mean ± SEM (n = 3) of each band is shown above each blot. *Significantly different from 0 μmol/L DIM, *P *< 0.05.

In order to determine whether the reduced cyclin A and increased p21^CIP1/WAF1 ^and p27^KIP1 ^protein expressions induced by DIM treatment resulted in the inhibition of CDK2 activity, total cell lysates were immunoprecipitated with the CDK2 antibody followed by *in vitro *kinase assays, using histone H1 as the substrate. A reduction in CDK2 activity was detected within 2 h after treatment with 30 μmol/L of DIM, with a significant difference becoming apparent during 12 h of DIM treatment (Fig. 4A).

Members of the Rb protein family are phosphorylated by CDKs, resulting in the activation of gene expression required for cell cycle progression. Because DIM inhibited CDK2 activity, we subsequently attempted to determine whether the reduction in CDK activity induced by DIM treatment led to reduced phosphorylation of the Rb protein. Western blot analysis of total cell lysates with phospho-Rb antibody revealed that phospho-Rb levels were reduced in the DIM-treated cells. When the immunoblot was probed using total Rb antibody, two bands were detected with an increase in the intensity of the lower band (hypophosphorylated Rb) being detected in the DIM-treated HT-29 cells, whereas the intensity of the higher band (hyperphosphorylated Rb) was reduced by DIM treatment. The levels of the E2F-1 protein were significantly lower in the cells treated with DIM for 12 h (Fig. 4B).

### DIM inhibits CDC2 activity

**Figure 5:**
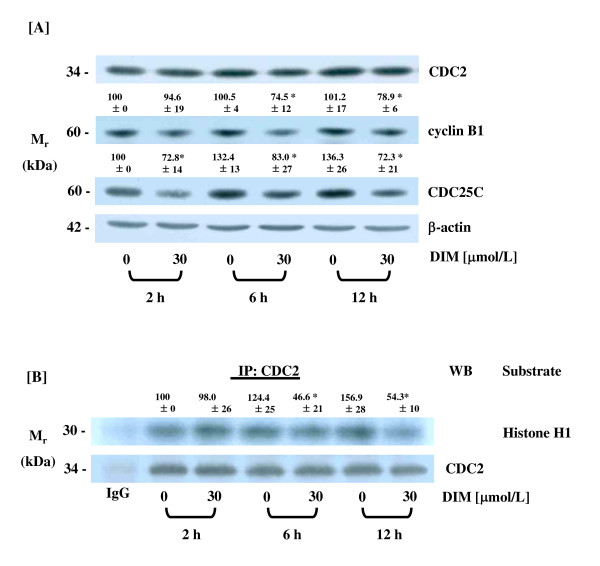
**DIM reduces the protein levels of cyclin B1 and CDC25C and the activity of CDC2 in HT-29 cells**. Cells were treated with different concentrations of DIM as described in Figure 1. (A) Cell lysates were analyzed via Western blotting with the indicated antibodies. Photographs of the chemiluminescent detection of the blots, which were representative of three independent experiments, are shown. (B) CDC2 was immunoprecipitated and analyzed by an *in vitro *kinase assay, using histone H1 as a substrate. Photographs of an autoradiograph of the dried gel, which are representative of three independent experiments, are provided. The relative abundance of each band to its own β-actin (A) or CDC2 (B) was quantified, and the control levels were set at 100%. The adjusted mean ± SEM (n = 3) of each band is shown above each blot. *Significantly different from 0 μmol/L DIM, *P *< 0.05.

To elucidate the mechanisms by which DIM induces G2/M arrest, the levels of G2/M regulatory proteins were assessed. The protein levels of cyclin B1 and CDC25C were reduced in cells treated with 30 μmol/L of DIM at 6 h and 2 h, respectively. DIM had no effect on the levels of the CDC2 protein (Fig. 5A). *In vitro *kinase assays were conducted to assess CDC2 activity after pulling down the CDC2 immune complex with CDC2 antibody. CDC2 kinase activity was reduced significantly at 6 h after the addition of DIM (Fig. 5B).

## Discussion

Preliminary clinical trials in women suggest that the I3C found in cabbage, broccoli, Brussels sprouts, and cauliflower is a promising agent against breast and cervical cancer (reviewed in [14]). I3C has been observed to reduce the development of cancer in animals when administered prior to or simultaneously with a carcinogen, whereas it enhanced the development of cancer when administered after carcinogen treatment (Reviewed in [5]). For example, Plate *et al*. reported that I3C reduced azoxymethane (AOM)-induced aberrant crypt foci formation in rats [37], whereas Suzui *et al*. observed that I3C increased the tumor multiplicity of adenocarcinomas in the colon of AOM-treated rats [38]. Because it is believed that DIM mediates the anticarcinogenic effects of I3C, it is important to understand the mechanisms by which DIM exerts its anticarcinogenic effects.

In our previous study, we demonstrated that DIM inhibits cell growth and induces apoptosis in HT-29 and HCT-116 human colon cancer cells in a concentration-dependent manner (10–30 μmol/L). However, unpublished results in our laboratories showed that I3C at the same concentrations did not alter the viable cell numbers of either HT-29 human colon cancer or DU145 human prostate cancer cells (Kim EJ and Park JHY, unpublished results). In the present study, using HT-29 cells, we have shown that 1) DIM induces G1 and G2/M arrest; 2) DIM reduces the protein levels of cyclin A, D1, and CDK4. 3) DIM increased the protein levels of the CDK inhibitors, p21^CIP1/WAF1 ^and p27^KIP1^; 4) DIM reduced the phosphorylation of Rb and the levels of E2F-1, as well as CDK2 activity; 5) DIM reduced the levels of CDC25C phosphatase and cyclin B1, as well as CDC2 activity. Hanahan and Weinberg [25] described six necessary changes in cell physiology that dictate malignant growth, two of which are the self-sufficiency of growth signals and the evasion of programmed cell death or apoptosis. Therefore, a bioactive compound such as DIM, which has the ability to induce apoptosis and inhibit cell cycle progression in colon cancer cells, may potentially be utilized as a chemopreventive agent for colon cancer. More studies with animal models and humans will be necessary to further determine whether DIM might potentially prove useful as an agent to prevent the development of colon cancer.

There is currently a dearth of data regarding the pharmacokinetics of DIM and its tissue distribution in humans [39]. Therefore, it is difficult to extrapolate the findings of the present study to humans. In this study, we utilized DIM at a concentration of 10 – 30 μmol/L, and Hong *et al*. [24] previously assessed the effects of 10 – 100 μmol/L DIM on cell cycle progression in breast cancer cells. We demonstrated, in a previous study, that DIM at concentrations of 10 – 30 μmol/L had no effect on the growth of IEC-6 cells, a normal intestinal epithelial cell line derived from the rat jejunum [31]. DIM was detected in the plasma (0.8 μg/mL or 3.24 μmol/L) and liver tissue (4 μg/mL or 16.3 μmol/L) of CD-1 female mice 2 h after the oral administration of 250 mg/kg of I3C [40]. In a phase I trial of I3C, DIM was reported to be the only detectable compound and reached a level of 607 ng/mL (2.46 μmol/L) after the administration of a single dose of 1000 mg [41]. Therefore, it is possible that the concentrations of DIM in the plasma and tissues of mice can be increased to the levels employed in our current study. It is also possible that low gut uptake of DIM may produce elevated DIM concentrations in the lumen of the colon, which can be made available to the colonocytes. It remains to be determined whether the concentrations of DIM utilized in the present study are clinically pertinent, by determining the concentrations of DIM in human plasma or the colonic lumen after the chronic administration of I3C and/or DIM.

The mammalian cell cycle is regulated by CDKs. CDK4 and 6 initiate the phosphorylation of Rb, which is augmented by the activity of CDK2 complexes with cyclins A and E. The Rb family proteins bind to members of the E2F transcription factor family and block the E2F-dependent transcription of genes controlling the G1 to S phase transition and subsequent DNA synthesis [42]. The phosphorylation of Rb disrupts its association with E2F, thereby reducing the suppression of E2F target genes (reviewed in [43]). In the current study, DIM drastically reduced the levels of CDK4 and cyclin D1 and increased the levels of p21^CI1/CIP1 ^and p27^KIP1 ^proteins, thus suggesting that CDK4 activity may have been reduced in the DIM-treated cells. We also noted that DIM inhibited CDK2 activity and Rb phosphorylation. In addition to the observed reduction in Rb phosphorylation, DIM induced a reduction in the levels of the E2F-1 protein. These findings indicate that the expression of Rb-E2F regulatory targets may have been reduced in DIM-treated cells, and this phenomenon may have contributed to DIM-induced G1 arrest in HT-29 cells.

p21^CIP1/WAF1 ^expression is usually controlled at the transcriptional level by both p53-dependent and -independent mechanisms [44]. In the present study, we noted that DIM induced p21^CIP1/WAF1 ^mRNA levels in HT-29 cells (Fig. 3), which harbor a mutant p53 gene [45]. In addition, in our previous study, we demonstrated that p53 levels were not affected by DIM treatment in HCT116 human colon cancer cells, which harbor the wild-type p53 gene [31]. These results show that DIM increases p21^CIP1/WAF1 ^levels via p53-independent mechanisms.

Entry into mitosis is regulated by the activity of the cyclin B/CDC2 complex, which is tightly controlled by its phosphorylation status. CDC2 is inactive in phosphorylated form, and the CDC25C phosphatase removes the phosphates of Thr14 and Tyr15 in CDC2, resulting in the activation of the cyclinB/CDC2 complex (Review in [46]). In the current study, DIM caused a reduction in the levels of cyclin B1 and CDC25C proteins, an effect which may have contributed to reduced CDC2 activity, ultimately leading to G2/M cell cycle delay.

## Conclusion

We have demonstrated that DIM inhibits HT-29 cell proliferation and induces G1 and G2/M phase arrest, both of which are associated with reduced CDK2 and CDC2 activity, respectively. The current results, together with the results of our previous work [31], show that DIM inhibits colon cancer cell growth via the induction of cell cycle arrest and apoptosis. Future animal and human studies will be required to determine whether DIM can be applied as a potential agent for the prevention of colon cancer.

## Abbreviations

DIM: 3,3'-diindolylmethane; CDK: cyclin-dependent kinase; Rb: retinoblastoma proteins; CDC: cell division cycle.

## Competing interests

The authors declare that they have no competing interests.

## Authors' contributions

HJC, DYL, and JHYP designed the project. HJC performed the overall biochemical analysis and wrote the first draft of the manuscript. DYL performed the CDK assays. JHYP directed the overall study and revised the manuscript. All authors contributed to the discussion of the data, and read and approved the final manuscript.

## Pre-publication history

The pre-publication history for this paper can be accessed here:

http://www.biomedcentral.com/1471-230X/9/39/prepub
